# How effective are international deployments in strengthening low- and middle-income countries (LMICs) to respond to outbreaks in the long term?

**DOI:** 10.1136/bmjgh-2025-022221

**Published:** 2026-01-27

**Authors:** Femi Nzegwu, Farhana Haque, Elizabeth Clery, Neema Kamara, Edouard Nkunzimana, Merawi Aragaw Tegegne, Edmund N Newman, Radjabu Bigirimana

**Affiliations:** 1 UK Public Health Rapid Support Team (UK-PHRST), Department of Infectious Disease Epidemiology and Dynamics, London School of Hygiene & Tropical Medicine (LSHTM), London, UK; 2 Emergency Preparedness and Response Division (EPR), Africa Centres for Disease Control and Prevention (Africa CDC), Addis Ababa, Ethiopia; 3 UK Public Health Rapid Support Team (UK-PHRST), UK Health Security Agency (UKHSA), London, UK

**Keywords:** Pandemic Preparedness, Interdisciplinary Research, Global Health, Africa, Health systems evaluation

## Abstract

**Introduction:**

International public health deployments are frequently used to support outbreak response, but there is limited evidence of their long-term impact on national response systems. This study assessed the extent to which deployments contribute to long-term, sustained impacts on the national outbreak response capacities of African Union Member States.

**Methods:**

We used an exploratory sequential mixed-methods design for this study. We conducted 83 key informant interviews across ten countries, carried out two in-depth country case studies and administered an online survey among 304 stakeholders involved in international deployments from 28 African Union Member States. Qualitative data were analysed thematically. Adjusted ORs (aORs) and 95% CIs identified factors associated with perceived long-term, sustained impact of deployments using multivariable logistic regression.

**Results:**

International deployments contributed to long-term impacts in national outbreak response across three domains: (1) strengthened systems and protocols; (2) continued use of infrastructure and equipment introduced during deployments and (3) enhanced confidence, knowledge and leadership among national stakeholders. Case studies further illustrated how adaptive, context-aware, collaborative deployments fostered national ownership and institutional memory. Deployments that were timely (aOR 4.4, CI 1.3 to 15.2), supported by deploying agencies (aOR 9.1, CI 2.1 to 39.9) and involved flexible and adaptive deployees (aOR 12.1, CI 1.9 to 77.1) were more likely to make substantial impact on national outbreak response.

**Conclusion:**

International deployments contribute to the sustained impact of outbreak response, particularly when they are country-led and align with local priorities. The findings suggest that international deployments should be viewed not only as emergency surge mechanisms, but also as strategic opportunities for contributing to longer-term impacts on national systems. Future deployment models should prioritise developing soft skills of deployees, ensure deployments are timely, context-appropriate and supported with additional resources to maximise their enduring value.

**WHAT IS ALREADY KNOWN ON THIS TOPIC**

- Considerable resources are invested for deploying international experts to provide short-term technical and/or operational support to control outbreaks and public health emergencies affecting resource-constrained countries globally.
- While international deployments are effective in delivering short-term outputs, there is limited evidence of their long-term impacts on national epidemic response systems.

**WHAT THIS STUDY ADDS**

- International deployments made sustained impacts on national response capacities by strengthening systems and protocols, building local expertise and leadership and through sustained use of infrastructure and equipment brought in during deployments.
- Several contextual and operational predictors that enabled sustained impact of international deployments were also identified.

**HOW THIS STUDY MIGHT AFFECT RESEARCH, PRACTICE OR POLICY**

- This study offers new evidence that international deployments are not merely emergency surge mechanisms but can also be leveraged to simultaneously strengthen national epidemic response capacities and systems.

## Introduction

 When public health emergencies occur across the world, specialist teams are deployed internationally to support countries. In this paper, we define international deployment as the placement of multidisciplinary specialist(s) in low- and middle-income countries (LMICs) to support the management of infectious disease outbreaks and/or epidemics. These teams come from a range of institutions—governments, regional bodies, international and national organisations.^1 2^ The models employed by these teams differ widely. Some are characterised by multidisciplinary skillsets and rapid deployability, often focusing on epidemic preparedness and response, sometimes with context-specific research, capacity sharing and learning happening either simultaneously or after the deployment. Other models of deployments target only specific aspects of an outbreak response such as surveillance. Deployment times can vary greatly from a few days to many months.^2^,^10^ Whichever method is adopted, the intent is to strengthen national capacity to safeguard populations from public health threats.^6 7 11 12^

In the UK and around the world, millions of pounds are spent every year on rapid deployments to support LMICs to avert or contain public health emergencies of national and/or international concern.^13 14^ The allocated budget for the Health Emergencies Programme of the World Health Organisation between 2022 and 2023 was estimated at US$1.25 billion.^15^ A considerable proportion of this funding was committed to sending short-term technical and/or operational experts to outbreak sites to assist with the epidemic response.

While the results of an international deployment are documented mainly through post-deployment debriefs and reports, there is limited understanding of the long-term, sustained impacts of these approaches.^416^,^19^ This study sought to understand the nature of any long-term impacts of international deployments, explore what, if any, difference is made to national capacities of countries receiving these deployments and understand any contextual or operational factors that may affect the perception of deployment impacts.

## Materials and methods

### Study design

We conducted an exploratory sequential mixed-methods study between July 2023 and April 2024 to assess whether and how international public health deployments contribute to long-term national outbreak preparedness and response systems. This design was selected to allow in-depth exploration of stakeholder experiences and perceptions using in-depth interviews and case studies (qualitative phase), followed by a quantitative survey to test associations more broadly.^20^,^22^ We hypothesised that international deployments contribute to sustained impacts when specific contextual and operational enabling conditions are present, including adequate pre-deployment preparation, appropriate and contextualised expertise and collaborative working with national teams.

### Study setting and participant selection

The study was implemented in the 28 African Union (AU) Member States that had received at least one international deployment from the Africa Centres for Disease Control and Prevention (CDC) and/or the UK Public Health Rapid Support Team (UK-PHRST) during 2020–2023. The Member States included Burkina Faso, Burundi, Cameroon, Chad, Democratic Republic of the Congo (DRC), Ethiopia, Eswatini, Gabon, The Gambia, Ghana, Lesotho, Liberia, Kenya, Madagascar, Malawi, Mali, Namibia, Nigeria, Rwanda, São Tomé and Príncipe, Sierra Leone, Somalia, Somaliland, South Africa, South Sudan, Tanzania, Uganda, Zambia and Zimbabwe. Participants included national and subnational health officials from the ministries of health and national public health institutes (NPHIs), incident managers, representatives of deploying agencies, individuals who were deployed (i.e., deployees) and civil society organisations involved in outbreak response. The deployees who participated in this study were based in 25 countries, namely Burundi, Burkina Faso, Cameroon, Chad, Democratic Republic of Congo, Eswatini, Ethiopia, Gabon, Ghana, Kenya, Lesotho, Liberia, Madagascar, Mali, Namibia, Nigeria, Rwanda, São Tomé and Príncipe, Sierra Leone, Somalia, South Africa, South Sudan, Uganda, United Kingdom and Zambia.

#### In-depth interviews

10 countries were purposively selected for the in-depth interviews to reflect a range of deployment levels.^21 23^ Deployment levels of countries were categorised as high, medium and low if a country received ≥16, 6–15 and ≤5 deployments between 2020 and 2023, respectively. Within these countries, key informants were identified purposively to ensure inclusion of individuals with direct and varied experiences of deployments, including national stakeholders, subnational health authorities, civil society representatives, deployees and deployment agency personnel.^21 23^

#### Case studies

Two countries (Namibia and Nigeria) were purposively selected for the case studies to provide an in-depth exploration of their deployment experiences.^20^ Namibia was selected as a country with a low level of deployment during the period examined and Nigeria as a country with a medium deployment level. Each case study included national and subnational health authorities, frontline health workers and deployment partners, ensuring multiple perspectives were fed into data collection and analysis.

#### Survey

The online survey targeted relevant stakeholders from 28 AU Member States that had received deployments in the timeframe. Purposive targeted sampling was used to recruit respondents likely to have direct knowledge and experience of disease outbreaks, deployment processes and impacts enabling a focused analysis on perspectives.^24^ The population sampled included: (1) individuals deployed internationally; (2) host-country staff who had engaged with deployed teams; (3) senior managers from deploying agencies.

### Data collection

We worked with a structured evaluation framework that considered three evaluation criteria (effectiveness, impact and sustainability) and seven study questions, which together provided the focus for all data collection and analysis (online supplemental appendix 1). We adopted the definitions of the three criteria from The Organisation for Economic Co-operation and Development (OECD) Development Assistance Committee evaluation criteria.^25^ We defined (1) effectiveness as the extent to which the international deployments achieved their objectives; (2) impact as the extent to which international deployments generated significant positive or negative, intended or unintended effects in country and (3) sustainability as the extent to which the net benefits of the work of these international deployments continued or are likely to continue well beyond the deployment. Gathering key lessons learnt was also an area of interest factored into the design. 10 thematic areas of deployment contribution guided our areas of enquiry—laboratory, epidemiology, surveillance and data management, infection, prevention and control (IPC), risk communication and community engagement, logistics, finance, psychosocial support/staff well-being and coordination (online supplemental appendix 2).

### Qualitative

#### In-depth interviews

Semi-structured interviews were conducted between July and November 2023, face to face and online (via Teams), lasting approximately 60–90 min. For the face-to-face interviews, participants were invited to the Africa CDC Headquarters in Addis Ababa. An interview guide, informed by early scoping interviews and a literature review, focused on deployment objectives, value, sustainability of contributions, enabling and limiting factors and country capacity for independent outbreak response. All interviews were audio recorded with informed consent and transcribed verbatim. All French interviews were translated into English for analysis.

#### Case studies

2-day participatory workshops were held in Namibia and Nigeria during the case studies, each with approximately 20 national health experts, to explore deployment experiences and national perceptions of impact in greater detail. The participatory workshops were jointly planned and co-facilitated by national experts from the NPHIs and Ministry of Health (MoH) and two study investigators (FN, FH). Data generated were summarised, reviewed and discussed on site by workshop participants to arrive at the final set of findings and recommendations. Key informant interviews with senior public health officials and a review of documentary sources (e.g., national emergency response plans, deployment reports, after-action reviews) supplemented the workshop findings.

#### Quantitative

A structured online questionnaire was designed in English and French using the RedCAP platform and administered between 1 July and 23 August 2024. The survey comprised 77 questions, including closed-ended questions on deployment characteristics, perceived long-term impact and contextual and operational factors, plus open-ended questions for additional narrative insights. Respondents were eligible if they had participated in or hosted an international deployment from 2020 to 2023. All French survey responses were translated into English for analysis.

### Sample size

Approximately 200 individuals were deployed to the Member States between 2020 and 2023. About 300 individuals from the Member States were involved in managing international deployees during the period. The required sample size for the survey was calculated based on the above population size of 500 stakeholders. Assuming a 95% CI and 5% margin of error, a sample size of at least 132 stakeholders was required to achieve 80% power for the online survey. This sample was proportionally allocated between the two stakeholder groups based on their relative sizes. We continued data collection for one-to-one, in-depth interviews until data saturation, often achieved between 10–12 interviews per stakeholder group.^26^

### Data Analysis

#### Qualitative

Interview and workshop transcripts were imported into NVivo (V.14) and analysed thematically following Braun and Clarke’s six-phase approach.^27^ Two researchers independently coded an initial set of transcripts to develop a codebook, resolving discrepancies by consensus. Themes were iteratively refined and defined, with illustrative participant quotes selected to highlight key findings. In vivo codes were retained where participant wording captured critical concepts.

Case study data were reviewed in real time for emergent themes which were then discussed for consensus with the workshop group participants. The participatory nature of the workshop shaped both data generation and interpretation. To ensure the centrality of participant voices, we retained in vivo codes where appropriate.

#### Quantitative

Survey data were exported from RedCAP into Stata/SE V.18.5 (StataCorp). Descriptive statistics summarised respondent demographics and deployment experiences. Associations between perceived long-term impact (sustained impact vs no or limited impact) and 21 independent variables reported by the survey respondents (that included both deployees and those involved in hosting or managing deployments) were tested using χ^2^ test.^28^ Variables with p<0.05 were entered into a multivariable binary logistic regression model. A backward elimination approach, which begins with all predictors and sequentially removes the least significant variable at each step, was used to produce a final model containing only variables contributing meaningfully to perceived impact. Adjusted ORs (aOR) and 95% CIs were reported. Model performance was evaluated using the Hosmer-Lemeshow goodness-of-fit test and area under Receiver Operating Characteristic (ROC) curve (AUC), and explained variance was assessed with McFadden’s Pseudo R².^29^

### Integration of findings

Qualitative and quantitative approaches were integrated during the methods and the interpretation stages using a convergent approach. Data from the qualitative phase were used to inform the data collection approach of the quantitative phase. Specifically, the findings from the qualitative phase informed the conceptualisation of the construct of interest and the development and piloting of the survey instrument. Several contextual, operational and individual characteristics identified during the in-depth interviews and case studies informed the development and refinement of the survey instrument. During the interpretation stage, quantitative findings identified significant statistical associations, while qualitative data explained the contextual and relational mechanisms underlying these associations.

### Patient and public involvement

It was not appropriate or possible to involve patients or the public in the design, conduct, reporting or dissemination plans of our research.

## RESULTS

### In-depth interviews

65 online and 18 face-to-face interviews were conducted with participants from 10 countries—Burkina Faso, Burundi, Cameroon, Malawi, Namibia, Nigeria, South Africa, Uganda, Zambia and Zimbabwe. Most of the participants were males (64%). Participants represented national health authorities including ministry of health officials, subnational health officials, partner organisations, civil society representatives and management of deploying organisations (table 1).

**Table 1:** Organisational affiliations and sex of interviewees

Employment/organisational affiliations	Female (%)	Male (%)	Number (%)
National health authorities	8 (10)	7 (8)	15 (18)
Subnational health authorities	3 (3)	10 (13)	13 (16)
National public health institutes	6 (7)	14 (17)	20 (24)
Civil society representatives	4 (5)	2 (2)	6 (7)
Deployees	4 (5)	8 (10)	12 (15)
Partner agencies	4 (5)	7 (8)	11 (13)
Deploying agencies	1 (1)	5 (6)	6 (7)
Total	30 (36)	53 (64)	83 (100)

#### Emergent themes

Three categories of perceived, long-term impacts emerged from the qualitative interviews. These included: (1) systems, protocols and processes available and accessible for future disease outbreaks; (2) physical infrastructure and equipment in place and used as is or repurposed to address future disease outbreaks and (3) knowledge of disease outbreak response retained, made more widely available and applied to future outbreaks.

#### Systems, protocols and processes in place for future disease outbreaks

Interviewees provided evidence that the systems, protocols and processes developed by deployees in response to specific disease outbreaks had been maintained and were being used, or were available for use, for other disease outbreaks. This was particularly relevant for improving co-ordination and surveillance systems. Focusing on the coordination aspect, one national stakeholder described how this had created impact:

COVID really helped with system strengthening. And with that, if we're to have something come now, we have a platform we can work on. I can start with coordination, even for a few days…because now we always work through the Public Health Emergency Centre. So, you have this unified command every time.

In terms of surveillance, an external stakeholder described its impact as follows:

If there was a specific gap…I think from our surveillance and analytics side a very, very clear one was we were having challenges within the Ebola response with data information systems and analytics. A deployment was made – a team which joined the Ministry of Health developed a document that enabled them to follow a specific method of doing analytics. And that has now become the standard of response analytics.

However, some national stakeholders occasionally noted that it was harder to sustain and further develop systems, processes and protocols, without the presence of international deployees as the quote below illustrates (translated from French):

We’ve kept these procedures, this way of doing things, these strategies that we developed together, we’ve kept them. But it’s important to note that it’s a bit difficult to maintain the same level when you have the support of partners and when you don’t. Because when we say partner support, not only is there technical support, but there is also financial support. There’s a whole range of things that follow on from that.

#### Physical infrastructure and equipment in place for future disease outbreaks

Stakeholders from many countries described how physical infrastructure constructed or installed by international deployees continued to be used in response to other disease outbreaks. One interviewee described how a mobile laboratory, deployable within 24 hours or as needed, and left behind by deployees from a foreign government (funder) was still in active use:

imagine where we were… now we have the capacity.

Some interviewees recounted how treatment centres constructed by some deploying agencies in response to a cholera outbreak were being used for other disease outbreaks. A respondent elaborated on this point:

If you take the COVID-19 model, which is the most recent model you have in many countries, I think that now in the country we have capacity in all the technology. We have a lab which has biomolecular capacity for example. This is something that in 2020 when COVID started was not the reality in the country.

In several instances, physical infrastructure constructed by international deployments had been re-purposed as this national interviewee explained:

The Ebola treatment units were built, I think about two. So, these are now the structures that the Ministry of Health is using to support trainings, similar trainings in terms of response and preparedness…they will be used as…centres of excellence for the Ministry of Health.

Other interviewees noted that, while long-term infrastructure had been created from international deployments, in some cases these were no longer used for their original purposes, although reconfiguration for other outbreaks was possible. There was some evidence that equipment purchased or supplied through an international deployment continued to be used, often for different purposes. An interviewee described how the equipment (including computers) installed in the COVID-19 vaccination centres, which had now been decommissioned, had been moved to the districts for use. In one country, the oxygen plants constructed by international deployees in hospitals in response to the COVID-19 outbreak were still in use for a range of other diseases.

Interviewees frequently described how they had encountered problems with continuing to use equipment supplied by international deployments, due to issues of maintenance and licensing.

#### Knowledge of disease outbreak response maintained and applied to future outbreaks

Almost universally, respondents indicated that their experiences of working with (and receiving training from) international deployees had equipped them with greater knowledge and confidence to respond to disease outbreaks. Many interviewees indicated that they had effectively used this increased knowledge and confidence in subsequent disease outbreaks as expressed by this national health official:

I’ve benefited a lot from these deployment in terms of capacity building, which has enabled me to manage the province well… which is my responsibility in terms of preparing for responses to epidemics.

More broadly, several interviewees outlined how the knowledge acquired by their colleagues through training had improved the team’s overall preparedness in relation to other disease outbreaks as expressed in this quote:

Today, our 13 regions can perform viral loads. We have staff trained in the incident management system. We have human resources who are now aware of the situation.

### Case studies

Case study participants identified three similar areas of the most impactful contributions made by international deployees, which remained in use beyond the specific outbreak they were created to address.

#### Systems, processes and products

These included enhanced surveillance systems, improved Public Health Emergency Operations Centre (PHEOC) management; development of ‘products’ such as Information, Education and Communication materials (e.g., for community awareness creation) and standard operating procedures for sample collection and transportation. A participant leading a national surveillance team explained it as follows:

They helped us to automate a system where once you enter all the data, you generate your charts, your map, and generate a Sitrep. And since then, we've been using that for every one of the outbreaks.

#### Equipment and infrastructure

These included setting up and equipping the PHEOC, improved laboratory networks in countries for improved disease detection, including reduced turnaround times for laboratory results as highlighted by the following quote from a leading national academic:

We continue to do that [run our labs now] on our own, because our system—how it was designed from the lab side—was not only to respond to COVID, but also to be used for diagnostic purposes, for surveillance purposes, for other infectious disease.

#### Learning and confidence building

These entailed the development of after-action reviews which provide detailed recommendations on how to improve subsequent responses and the development of enabling capacities in specific technical sectors (such as improved case management and IPC). Below is a quote from a senior executive from a global public health agency on the focus by international deployments on IPC during the COVID-19 pandemic which led to great advancements in this sector for the country:

“Where, with the support of the deploying agency in convening expertise, bringing in colleagues from within the continent and beyond the continent to work with us… we further developed our own competence…And now there are clear outputs: we have a new IPC model, we’re driving the development of IPC across the public health ecosystem. This is influential work, not only in outbreak response, but in more longer-term issues like antimicrobial resistance and things like that.

Ensuring that countries were part of international networks where peer-to-peer learning could occur was also seen as an important factor in creating long-term impact as this quote from a national public health leader within a ministry illustrates:

From our side, what we’ve done with most of the people who came to our lab, who we have interacted with, who have helped us—we have kept contacts, and we always write to them if we have issues and say do you know someone who can help us with this? There’s more like what we call a community of practice.

Consistently highlighted across both countries as facilitators of long-term impact were increased confidence, knowledge and skills of country-based staff and long-term improvements to the quality and coverage of existing public health systems, especially by ensuring rapid follow-up on gaps identified during the deployment.

Interviewees described how the experience of working alongside international deployees had increased their own knowledge in relation to outbreak response and thus their confidence in dealing with similar outbreaks, independently, in the future—illustrated in the quotes from directors of NPHIs below:

Having worked together with national and international teams, we’re more confident in our work. We seem ready, more prepared than we were in the past. So, I think that it is all because of the experience of standing shoulder to shoulder against these common outbreaks and having learned from each other.

One aspect that I would also like to mention is that feeling…that confidence that the country gained, you know that…I think it’s a soft skill, it’s not easily measurable, but you feel it, that the country feels like we have been empowered, and we are able to take charge.

Addressing gaps identified during an international deployment after the deployment was a theme that strongly resonated across both countries, highlighting the need for ongoing technical and financial assistance post-deployment. These were seen as crucial contributors to ongoing learning and sustainability. This is captured eloquently by the following quote from a former NPHI director:

You do the initial response…let’s take diphtheria. You recognise there’s a deficit in diagnostics, for instance. The six weeks [deployment] leads to an identification of a gap. You then agree with the international deploying agency: Listen, let’s do a longer deployment to support us to develop our capabilities in that area of deficit, in diagnostics. Then someone else comes following the initial six weeks for a three month deployment to support the organisation to build capacity. So next time, there’s not only ‘have we responded to the outbreak’—we identified a gap, we addressed the gap, and through that a relationship is built. And through that, people get to know each other and exchange learning, continue to work together in different ways that are mutually beneficial—they can end up publishing, they can end up developing new guidelines…But that way you’re developing…and of course the deployees benefit from that…So that’s the type of relationship that will have to happen…

### Survey

**Figure 1:**
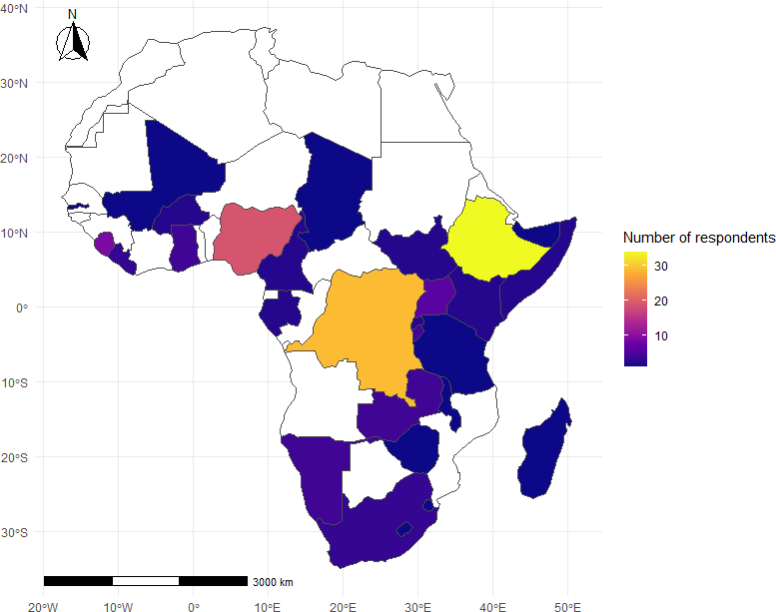
Map showing the number of survey respondents representing the African Union Member States.

The survey included a total of 304 respondents representing a broad range of African countries, with the largest proportions from Nigeria (19%), DRC (17%) and Ethiopia (16%). Organisational affiliations included Africa CDC (33%), ministries of health (28%), other government departments (17%) and NGOs (10%). Most respondents were experienced, with 28% having >10 years of deployment experience, 34% with 5–10 years, 15% having 3–5 years and 10% having <3 years. The majority were males (78%) and 31–50 years of age (85%). In terms of roles, 53% (161/302) had both hosted and been deployed, 31% had hosted deployments only, and 16% had been deployed exclusively. Among the respondents, 143 (47%) completed all substantive sections of the survey relevant to the impact analysis. Among these, the majority (17.5%) worked in Ethiopia (figure 1), most were males (77%) and had 5–10 years of deployment experience (39%). 82% of the respondents reported that the objectives of the international deployments were fully met and 85% (121/143) of the respondents perceived that international deployment made sustained impacts on the national public health emergency response systems.

#### Bivariate associations

We conducted χ^2^ tests to examine associations between perceptions of long-term, sustained impact and several contextual and operational variables (table 2). 11 variables showed significant associations (p<0.05) with perceived sustained impact of deployment, including timeliness of deployments (p<0.001), support from deploying agencies (p<0.001) or incountry partners (p<0.001) and briefing quality (p=0.029). In addition, several deployee traits were also associated with perceived impact including deployees’ engagement with country teams as equal partners, their flexible and adaptive attitude, appropriateness of their skills, acknowledgement of national contributions, good understanding of country’s working environment and context, valuing country team’s expertise and the comfort level of deployees in approaching country teams (table 2).

**Table 2:** Association between contextual/operational conditions and perceived sustained impact of international deployments (χ^2^ test)

Predictor variable	Sustained impact	No/limited impact	χ²	P value
Contextual understanding				
Understood country context	12	105	5.834	**0.016**
Understood organisation context	9	108	1.647	0.199
Preparation				
Clear understanding of Terms of Reference (ToR)	5	112	1.155	0.282
Appropriately briefed	10	107	4.742	**0.029**
Adequately prepared	12	105	1.572	0.21
Expertise, attitude, collaboration, cultural competence and humility				
Acknowledged national contributions	11	105	9.673	**0.002**
Worked collaboratively	3	114	0.358	0.55
Comfortable approaching country team	9	107	5.491	**0.019**
Country team comfortable questioning	11	107	0.605	0.437
Skills appropriate to needs	8	109	6.56	**0.01**
Listened/responded appropriately	9	108	3.013	0.083
Flexible/adaptive to needs	4	112	4.663	**0.031**
Valued country expertise	6	111	5.582	**0.018**
Respectful/helpful/capable	3	114	2.686	0.101
Engaged as equal partners	5	110	14.979	**<0.001**
Support and timing				
Deployments were timely	21	96	16.518	**<0.001**
Deployee supported by country partners	17	100	12.622	**<0.001**
Deployee supported by deploying agency	7	110	25.208	**<0.001**
Respondent demographics				
Gender (Male vs Female)	25	94	0.592	0.442
Experience (>5 years vs <5 years)	32	87	1.098	0.295
Age (>50 years vs 21–50 years)	18	102	0.44	0.507

#### Multivariable analysis

A multivariable binary logistic regression model was then fitted using the 11 statistically significant variables from the χ^2^ test as independent predictors, with perceived sustained impact as the dependent variable. Deployments that were timely (aOR 4.4, CI 1.3 to 15.2), supported by deploying agencies (aOR 9.1, CI 2.1 to 39.9) and involved flexible and adaptive deployees (aOR 12.1, CI 1.9 to 77.1) were more likely to make substantial perceived impact (table 3). The final multivariable logistic regression model showed good calibration (Hosmer-Lemeshow χ² = 27.35, p<0.0001) and acceptable discrimination, with an AUC of 0.797. The model demonstrated excellent fit, with a McFadden’s pseudo R² of 0.262.

**Table 3:** Multivariable logistic regression of factors associated with perceived substantial impact of international deployment

Variable	Adjusted OR (aOR)	95% CI	P value
Deployments were timely	4.4	1.3 to 15.2	0.020
Deployee flexible and willing to adapt	12.1	1.9 to 77.1	0.008
Deployee supported by deploying agency	9.1	2.1 to 39.9	0.003

## DISCUSSION

This study explored the perceived long-term impacts of international public health deployments on national and subnational outbreak response systems in multiple African countries. Drawing on an exploratory sequential mixed-methods approach—including in-depth interviews, two detailed case studies and a multicountry survey—we identified and triangulated key factors that underpin long-term impactful deployments. Our findings suggest that international deployments can, under the right enabling conditions, contribute to long-term impacts in systems, infrastructure and human capital. However, the sustainability of these impacts appears contingent on how deployments are structured and enacted, rather than solely on their technical content.

The quantitative findings suggest that deployments that were timely and associated with enabling conditions—additional support provided from deploying agencies and those that involved deployees who were flexible and adaptive to the country’s needs—were significantly associated with perceived long-term impact. Qualitative data also reinforced these findings by illustrating the value of timeliness, deploying agency support and appropriate behaviours and attitude of deployees. Our finding that perceived sustained impact was closely tied to whether deployees publicly acknowledged and promoted national contributions highlights the central role of equitable partnership dynamics in successful epidemic response support. Visible recognition of national expertise strengthens local ownership, legitimacy and trust, all of which are critical for translating short-term technical assistance into long-term system strengthening. Evidence from global health research consistently shows that partnerships characterised by mutual respect, shared credit and joint visibility foster stronger institutional capacity, greater motivation among local actors and more durable uptake of improved practices.^30^,^32^

A central theme to emerge was the embedding of systems, protocols and procedures established during international deployments into routine national operations for sustained systems strengthening and institutional memory. Participants consistently described how coordination structures and surveillance systems originally set up for specific outbreaks were repurposed and institutionalised. This supports the view that emergency response activities can lead to greater opportunities for system strengthening when structured intentionally.^33^ However, some respondents noted that the absence of ongoing technical and financial support post-deployment can challenge the sustainability of these systems—highlighting the importance of long-term partnership, not just short-term presence.

Another clear perceived impact was the continued use of infrastructure and equipment introduced through international deployments during specific outbreaks that were not only still in use but had been adapted for broader disease surveillance and case management. Yet this benefit was occasionally offset by concerns about maintenance and underutilisation—issues that echo the broader literature on vertical programming and the risks of funder-driven investment in infrastructure that lacks integration or local ownership.^34^,^36^ This reinforces the argument that infrastructure investments must be coupled with capacity-strengthening and governance arrangements to ensure functionality and relevance over time.^37^

The soft and intangible legacies of deployments including knowledge exchange, confidence building and leadership capacity emerged as major areas of contribution. Participants often described how joint response activities increased local technical competence and self-efficacy—both individually and institutionally. These soft impacts, though harder to quantify, were repeatedly highlighted in the case study countries and in our quantitative analysis and align with earlier evaluations which note that confidence and familiarity with structured response processes are key enablers of rapid, autonomous future action.^38 39^

Quantitative analysis using multivariable logistic regression identified several significant predictors of perceived sustained impact. Notably, the timeliness of deployment, flexibility and adaptive attitude of deployees and support from deploying agencies were all statistically associated with the perception of long-term impact. These findings reinforce the notion that how a deployment is conducted—its timeliness, preparation, alignment with local systems and interpersonal dynamics—can be as important as what it delivers. These factors likely operate through multiple pathways: timeliness enables early integration with national systems, preventing duplication and fostering trust; flexibility and adaptive attitudes facilitate co-creation and responsiveness to local priorities, strengthening absorptive and adaptive capacities within health systems. These findings align with resilience frameworks, which emphasise that health systems’ ability to absorb shocks and adapt depends on collaborative governance and context-sensitive interventions.^40^ The strong association between flexible and adaptive attitudes of deployees and perceived impact invites further exploration to determine whether these differences reflect interpersonal dynamics or working styles. The findings are indicative of growing evidence that inclusive, empathetic and collaborative approaches yield stronger outcomes in crisis settings.^38 41 42^

The enabling conditions highlighted by respondents—such as context-sensitive briefing, cultural competence, a posture of humility and mutuality and clear terms of reference—echo global guidance on effective technical assistance and localisation.^43 44^ These findings suggest that deployments should be framed not only as surge mechanisms but also as opportunities to model collaborative, respectful and reciprocal partnership.

A key strength of this study is its mixed-methods design, which combined qualitative and quantitative approaches and triangulated findings through case studies, providing both breadth and depth of insight. The purposive sampling strategy, while limiting statistical generalisability, was a deliberate strength in targeting individuals with direct deployment experience and informed perspectives on long-term impact. The application of inferential statistics, including χ^2^ test and logistic regression, allowed the identification of statistically significant predictors of sustained perceived impact, moving the analysis beyond descriptive insights. Finally, the involvement of both national and international respondents ensured a balanced perspective, reducing the potential for one-sided interpretation and increasing the relevance of findings for global health practice.

A few limitations are also noted. First, data relied on self-reported perceptions, which may be affected by recall bias or social desirability bias. Second, although the survey reached 304 respondents, only 47% completed all impact-related questions, and some subgroups were small. Although three factors were statistically significant predictors of perceived long-term impact, the CIs for some of these effects were wide. These wide CIs were most likely due to small subgroup sizes and sparse data for some predictors. The overall direction of effect was consistent, indicating that these factors were likely important contributors to sustained impact, but the exact magnitude of association may be uncertain, and results should be interpreted with caution. Qualitative findings supported these associations, highlighting how timely deployments, additional support from deploying agencies and the flexible and adaptive nature of deployees can foster trust, confidence and sustainability of outbreak response gains. Finally, as an observational study, we cannot infer causality; rather, we identify enabling conditions perceived to contribute to sustained impact. Future research would benefit from quasi-experimental designs or longitudinal data to assess impact over time, explore causal pathways and examine more objective outcome/impact indicators to validate these associations and move beyond perception-based metrics.

## Conclusions

This study offers new evidence on the long-term impacts of international deployments on strengthening national public health capacities and systems—an area often underexplored in emergency response evaluation. International deployments can deliver lasting impacts to outbreak preparedness and response systems, especially when relational and contextual enablers are present. This suggests that deployments should be treated as strategic opportunities for long-term system strengthening to maximise their long-term value. The findings have important implications for deployment planning and policy. International deployments should be regarded not only as emergency surge mechanisms but also as opportunities for strategic health system strengthening that can create long-term impacts for countries’ capacities to more readily and effectively respond to outbreaks. To enhance long-term impacts, future deployment strategies should place greater emphasis on cultivating deployees’ soft skills and ensure that deployments are context-sensitive, collaborative, timely and supported by appropriate resources. Future deployment models could formalise dual objectives: delivering rapid outbreak response while explicitly investing in longer-term national capacity and resilience, especially immediately post-deployment.

## Supplementary material

10.1136/bmjgh-2025-022221 online supplemental file 1

10.1136/bmjgh-2025-022221 online supplemental file 2

## Data Availability

Data are available upon reasonable request.

## References

[1] Greiner AL, Stehling-Ariza T, Bugli D (2020). Challenges in Public Health Rapid Response Team Management. Health Secur.

[2] Stehling-Ariza T, Lefevre A, Calles D (2017). Establishment of CDC Global Rapid Response Team to Ensure Global Health Security. *Emerg Infect Dis*.

[3] Aitken P, Leggat P, Robertson A (2009). Health and safety aspects of deployment of Australian disaster medical assistance team members: results of a national survey. Travel Med Infect Dis.

[4] Hamilton ARL, Södergård B, Liverani M (2022). The role of emergency medical teams in disaster response: a summary of the literature. *Nat Hazards*.

[5] Mackenzie JS, Drury P, Arthur RR (2014). The global outbreak alert and response network. Glob Public Health.

[6] Raftery P, Hossain M, Palmer J (2021). An innovative and integrated model for global outbreak response and research - a case study of the UK Public Health Rapid Support Team (UK-PHRST). BMC Public Health.

[7] Raftery P, Hossain M, Palmer J (2022). A conceptual framework for analysing partnership and synergy in a global health alliance: case of the UK Public Health Rapid Support Team. Health Policy Plan.

[8] Whitty CJM, Farrar J, Ferguson N (2014). Infectious disease: tough choices to reduce Ebola transmission. Nature New Biol.

[9] MSF (2020). Discover how we deliver medical humanitarian assistance.

[10] Christensen R, Fisher D, Salmon S (2021). Training for outbreak response through the Global Outbreak Alert and Response Network. BMC Med.

[11] World Health Organization (2024). WHO Implementation Guidance on Emergencies Capacity-Building: Approaches for Just-in-Time Learning Response to Health Emergencies.

[12] World Health Organization (2019). Health Emergency and Disaster Risk Management Framework.

[13] Jain V (2020). Financing global health emergency response: outbreaks, not agencies. J Public Health Policy.

[14] Xu M, Benn C, Reid-Henry S (2023). Rethinking international financing for health to better respond to future pandemics. BMJ Glob Health.

[15] WHO (2024). Programme budget. https://www.who.int/about/accountability/budget#:~:text=Emergency%20operations%20and%20appeals%20that,as%20the%20COVID%2D19%20pandemic.

[16] Hurtado C, Meyer D, Snyder M (2018). Evaluating the frequency of operational research conducted during the 2014-2016 West Africa Ebola epidemic. Int J Infect Dis.

[17] Jephcott FL (2024). Stuck in “the field”: why applied epidemiology needs to go home. BMJ Glob Health.

[18] Warsame A, Blanchet K, Checchi F (2020). Towards systematic evaluation of epidemic responses during humanitarian crises: a scoping review of existing public health evaluation frameworks. BMJ Glob Health.

[19] Shin YA, Yeo J, Jung K (2018). The Effectiveness of International Non-Governmental Organizations’ Response Operations during Public Health Emergency: Lessons Learned from the 2014 Ebola Outbreak in Sierra Leone. Int J Environ Res Public Health.

[20] Crowe S, Cresswell K, Robertson A (2011). The case study approach. BMC Med Res Methodol.

[21] Palinkas LA, Horwitz SM, Green CA (2015). Purposeful Sampling for Qualitative Data Collection and Analysis in Mixed Method Implementation Research. Adm Policy Ment Health.

[22] Shiyanbola OO, Rao D, Bolt D (2021). Using an exploratory sequential mixed methods design to adapt an Illness Perception Questionnaire for African Americans with diabetes: the mixed data integration process. Health Psychol Behav Med.

[23] Etikan I, Musa SA, Alkassim RS (2016). Comparison of Convenience Sampling and Purposive Sampling. *AJTAS*.

[24] Memon MA, Thurasamy R, Ting H (2024). PURPOSIVE SAMPLING: A REVIEW AND GUIDELINES FOR QUANTITATIVE RESEARCH. *JASEM*.

[25] DAC Evaluation criteria. https://www.oecd.org/en/topics/sub-issues/development-co-operation-evaluation-and-effectiveness/evaluation-criteria.html%20(2021.

[26] Guest G, Bunce A, Johnson L (2006). How Many Interviews Are Enough?: An Experiment with Data Saturation and Variability. Field methods.

[27] Braun V, Clarke V (2006). Using thematic analysis in psychology. Qual Res Psychol.

[28] Kim HY (2017). Statistical notes for clinical researchers: Chi-squared test and Fisher’s exact test. Restor Dent Endod.

[29] Jr (2013). Applied Logistic Regression.

[30] Alemu K, Berhanu D, Bergström A (2025). Towards equitable partnerships in global health research: experiences from Ethiopia, Uganda, Lao PDR and Vietnam. BMJ Glob Health.

[31] Harrison W, Gyapong M (2025). Building authentic partnerships across regions and disciplines to overcome global health threats. Int Health.

[32] Prasad S, Kamaara E, Compton B (2024). From short-term engagements to meaningful and equitable global health partnerships. PLOS Glob Public Health.

[33] Gooding K, Bertone MP, Loffreda G (2022). How can we strengthen partnership and coordination for health system emergency preparedness and response? Findings from a synthesis of experience across countries facing shocks. BMC Health Serv Res.

[34] Marks IH, Thomas H, Bakhet M (2019). Medical equipment donation in low-resource settings: a review of the literature and guidelines for surgery and anaesthesia in low-income and middle-income countries. BMJ Glob Health.

[35] Reddy M, Samprathi M, Bhatia V (2022). Medical Equipment Donation: An End in Itself or a Mean to an End?. Indian J Crit Care Med.

[36] Williams DB, Kohler JC, Howard A (2020). A framework for the management of donated medical devices based on perspectives of frontline public health care staff in Ghana. Med Access Point Care.

[37] OECD (2019). OECD/IMF Reference Note on the Governance of Quality Infrastructure Investment.

[38] Cooper S, Endacott R, Cant R (2010). Measuring non-technical skills in medical emergency care: a review of assessment measures. Open Access Emerg Med.

[39] Westman A, Kurland L, Hugelius K (2024). Non-technical skills needed by medical disaster responders- a scoping review. Scand J Trauma Resusc Emerg Med.

[40] Dsouza SM, Katyal A, Kalaskar S (2024). A scoping review of health systems resilience assessment frameworks. PLOS Glob Public Health.

[41] Barbanti Oj Global partnerships and development: beyond intractability. https://www.beyondintractability.org/essay/partnership-and-conflict.

[42] Power N, Alcock J, Philpot R (2024). The psychology of interoperability: A systematic review of joint working between the UK emergency services. J Occupat & Organ Psyc.

[43] (2021). IASC guidance on strengthening participation, representation and leadership of local and national actors in iasc humanitarian coordination mechanisms. https://interagencystandingcommittee.org/sites/default/files/migrated/2021-07/IASC%20Guidance%20on%20Strengthening%20Participation%2C%20Representation%20and%20Leadership%20of%20Local%20and%20National%20Actors%20in%20IASC%20Humanitarian%20Coordination%20Mechanisms_2.pdf.

[44] WHO Towards a Meaningful Engagement of Local and National Actors in the Health Cluster: Health Cluster Localization Strategy. 2024.

