# Supplementary material for: How effective are international deployments in strengthening low- and middle-income countries (LMICs) to respond to outbreaks in the long term?
Source: BMJ Glob Health. 2026 Jan 27;11(1):e022221. doi: 10.1136/bmjgh-2025-022221 (PMC12853481; doi:10.1136/bmjgh-2025-022221)
Supplement: online supplemental file 1 [file bmjgh-11-1-s001.docx]

**Appendix 1**: **Detailed study questions are as follows:**

| **Criteria** | **Example areas of enquiry** |
| --- | --- |
| Effectiveness | - How well did the deployment achieve their objectives? - What is the perceived value of the deployments? |
| Impact & sustainability | - What difference – positive and negative – have deployments created or enabled for the countries of deployment? Did this differ by gender and region? - How did these changes occur? - Are these changes sustainable? Are they embedded in structures and processes that allow for them to become routine ways of working? |
| Learning | - What are the factors (barriers and enablers) that affect deployments? - What are the key lessons learned from this study? |

| **Appendix 2: Matrix of thematic areas of contribution, potential outcomes and indicators measures** | | | |
| --- | --- | --- | --- |
| **Area of contribution** | **Outcomes** | **Potential indicators** | **Data collection methods** |
| 1. **Laboratory** | Enhanced laboratory capacity/Operational readiness | Lab guidance/protocols/ SOPs developed during the deployment or through international support that are being used in subsequent outbreak response | Surveys  Interviews |
|  |  | Lab training conducted with international support |  |
|  |  | Staff trained with international support provide training/share expertise to national staff (ToTs) |  |
|  |  | Provision & use of equipment, materials, infrastructure, mobile laboratories etc through international support |  |
| 1. **Epidemiology** | Enhanced capacity for outbreak detection, response and prevention | Local teams trained via international support able/ready/prepared to detect, investigate and respond to outbreaks | Surveys  Interviews |
|  |  | Staff trained with international support provide training/expertise to national staff (ToTs) |  |
|  |  | Trained staff are able to conduct operational research for improved outbreak response and prevention |  |
| 1. **Surveillance & data management** | Existence of a new or improved national surveillance system for emerging diseases | Staff competence in surveillance is improved through international support | Surveys  Interviews |
|  |  | Developed/reviewed and updated surveillance/ SOPs/guidelines through international support |  |
|  |  | A new or improved repository of surveillance data exists through international support |  |
|  |  | Surveillance action identified and implemented through international support |  |
|  | Enhanced data analytics demonstrated by the surveillance unit in its reporting | National surveillance staff trained through international support |  |
|  |  | Trained staff are able provide training/expertise to national staff (ToTs) |  |
|  |  | Application of skills, approaches, software acquired through international support in routine work |  |
| 1. **Infection Prevention and Control (IPC)** | Improved IPC practices for outbreak control and prevention | IPC guidelines/strategies/SOPs developed through international support | Surveys  Interviews |
|  |  | Local teams trained on improved IPC guidelines /strategies/SOPs through international support |  |
|  |  | Staff trained with international support provide training/expertise to national staff (ToTs) |  |
|  |  | Improved IPC guidelines /strategies/SOPs incorporated into existing practice |  |
| 1. **Risk Communication and community engagement** | Enhanced mechanisms in place to enable affected populations to make informed decisions to mitigate the effects of an outbreak | Evidence of a systematic approach in place to communicate to the entire community/population – e.g. protocols, planning guides, responsibilities etc. – through international support | Surveys  Interviews |
|  |  | Trained communication experts through international support who know how to use approach available. |  |
|  |  | Evidence that trained staff can and do apply a wide range of risk communications knowledge and practices. |  |
| 1. **Clinical case management** | Improved clinical case management | Orientation of clinical staff to outbreak response situations through international support | Surveys  Interviews  Case studies |
|  |  | Case management protocol developed through international support |  |
|  |  | Case management protocols incorporated/adopted locally through international support |  |
|  |  | Local teams trained on improved clinical guidelines through international support |  |
|  |  | Staff trained with international support provide training/expertise to national staff (ToTs) |  |
| 1. **Logistics** | Enhanced logistics system in place | Logistics procedures/ protocols available through international support | Case studies |
|  |  | Trained staff through international support |  |
|  |  | Staff trained with international support provide training/expertise to national staff (ToTs) |  |
|  |  | Staff can apply protocols to effectively and efficiently support procurement and supply chain management; asset and equipment management with international support |  |
| 1. **Finance** | Enhanced operational mechanism for disbursing funds as required | Established budget to support preparedness and response operations (i.e. salary, per diem, travel, equipment, vaccinations, database maintenance, etc.) with international support | Case studies |
|  |  | Provision of funds/other resources for managing outbreaks through international support |  |
|  |  | Rapid release of funds as required through international support |  |
| 1. **Psychosocial support/staff wellbeing** | Mechanisms to ensure RRT safety, health and wellbeing in place. | Evidence of prompt salary payment mechanisms through international support | Surveys  Interviews  Case studies |
|  |  | Evidence of medical/disability                   /life insurance, medical care, mental health care, emergency evacuation etc. system in place with international support |  |
| 1. **On the ground coordination** | Coordinated response of both national and international expertise | Effective mechanism for clearly delineating, allocating and implementing tasks are operational with international support. | Case studies |
