# Supplementary material for: How effective are international deployments in strengthening low- and middle-income countries (LMICs) to respond to outbreaks in the long term?
Source: BMJ Glob Health. 2026 Jan 27;11(1):e022221. doi: 10.1136/bmjgh-2025-022221 (PMC12853481; doi:10.1136/bmjgh-2025-022221)
Supplement: online supplemental file 2 [file bmjgh-11-1-s002.docx]

**Appendix S3 ─ Reflexivity Statement**

**How effective are international deployments in strengthening low- and middle-income countries (LMICs) to respond to outbreaks in the long-term?**

**Study conceptualization**

(1) How does this study address local research and policy priorities?

This study was conducted in the 28 countries within Africa that were affected by varying degrees of public health emergencies including infectious disease outbreaks between 2020 and 2023. The reduction of outbreaks in Africa is both a national, regional and global priority. Infectious diseases pose a significant threat to the continent's health and economic development; and to global stability with the risk of new pandemics emerging from Africa being a primary concern. The Africa Centres for Disease Control and Prevention (Africa CDC), the World Health Organization (WHO), the U.S. Centers for Disease Control and Prevention (CDC) and international partnerships including the UK Public Health Rapid Support Team (UK PHRST) are playing a crucial role in providing short-term surge capacity for tackling outbreaks in Africa by deploying rapid response teams as part of their strategies for mitigating the impact of outbreaks on the continent. Research into identifying the long-term impact, strengths and weaknesses of the existing international support mechanisms including deployments have become top priorities for the Member States as well as the deploying agencies, international partners and funders to build sustainable systems.

(2) How were local researchers involved in study design?

African researchers (Dr Radjabu Bigirimana, Dr Merawi Aragaw, Ms Neema Kamara and Mr Edouard Nkunzimana) were central in defining the research questions, identifying relevant contexts, and co-developing the study protocol to ensure national and regional appropriateness and policy relevance. Dr Bigirimana who leads the African Volunteers Health Corps at Africa CDC (the key mechanism for deploying rapid responders within Africa) and UK researchers, Dr Nzegwu and Dr Haque (who are members of the UK PHRST involved in deploying rapid responders from the UK) with support from other co-investigators co-developed and co-implemented this study to evaluate the impact of international deployments to enhance their sustainability and effectiveness.

**Research management**

(3) How has funding been used to support the local research team(s)?

Funding was shared equitably between the two key partner organisations involved in the study – Africa CDC and UK PHRST. Resources were jointly allocated to support in-country activities such as convening research meetings, workshops and related activities in national and regional venues. All staff salaries were borne by their respective employing institutions.

**Data acquisition and analysis:**

(4) How are research staff who conducted data collection acknowledged?

All investigators have been included as co-authors, and given priority as joint first authors, senior author and the subsequent authors to reflect their contributions to the study. Contributions made by the data collection team have been recognised in the acknowledgment section of the paper.

(5) How have members of the research partnership been provided with access to study data?

All members of the research partnership had equal access to the study data through a secure shared platform, ensuring transparency and joint ownership. Coinvestigators with relevant expertise worked with these datasets to run analysis. All authors requested analyses that satisfied their individual probing of the data for consistency and completeness.

(6) How were data used to develop analytical skills within the partnership?

The lead investigators arranged multiple meetings with other team members within the partnership for building necessary skills. Additional trainings for individual team members on statistical and thematic analyses were sought from relevant methodological experts from the London School of Hygiene and Tropical Medicine where necessary. All researchers were actively involved in data analysis, reflection, and discussion through face-to-face and online workshops to enhance skills and ensure balanced contributions to interpretation.

**Data interpretation**

(7) How have research partners collaborated in interpreting study data?

We used an iterative, participatory approach throughout the study where all researchers were jointly involved in the conception, design, data acquisition, data analysis and data interpretation. Data interpretation was carried out collaboratively during multiple structured online and four in-person workshops/meetings, ensuring that contextual insights and technical experts from both high-income and low-income countries shaped conclusions jointly. The first in-person and two online workshops focused on defining the research issue and ensuring equitable partnerships. A core working group composed of African and UK based researchers was formed to conduct a literature review. The group met online monthly for three months to ensure collaborative interpretation of the existing literature that guided the subsequent data collection using qualitative and quantitative methods. Following the in-depth interviews, the core group of researchers met in-person in Addis Ababa and then online biweekly to jointly analyse and interpret the data with the help of a qualitative data analyst in the team. Findings were validated further with all researchers and local stakeholders during the two in-person workshops in Nigeria and Namibia. The qualitative findings guided the quantitative survey. Integration of the qualitative and quantitative findings were conducted jointly via multiple online meetings. Finally, the findings were disseminated with all researchers and stakeholders involved in the study in an online validation workshop.

**Drafting and revising for intellectual content**

(8) How were research partners supported to develop writing skills?

Given that all co-authors were skilled in manuscript writing, specific workshops to develop writing skills were not conducted. Drafting and revisions were undertaken jointly throughout the study.

(9) How will research products be shared to address local needs?

As described earlier, the preliminary study results have been disseminated among all stakeholders involved with the study via a two-day, online validation workshop. Final products will be disseminated not only through open access, international peer-reviewed journals but also via local stakeholder meetings, policy briefs, and presentations to ministries of health, relevant development partners, deploying agencies and funders to ensure accessibility and local as well as international impact.

**Authorship**

(10) How is the leadership, contribution and ownership of this work by LMIC researchers recognised within the authorship?

This paper is coauthored by four LMIC researchers and four UK researchers reflecting equal ownership of the study. LMIC author Radjabu Bigirimana worked as the senior author for this paper and LMIC authors Neema Kamara, Edouard Nkunzimana and Merawi Aragaw were co-authors in this paper. LMIC team members were given ample opportunity to sign off on the final manuscript version. All authors agreed to be accountable for all aspects of the work in ensuring that questions related to the accuracy or integrity of any part of the work are properly investigated and resolved.

(11) How have early career researchers across the partnership been included within the authorship team?

We have included early career researchers from both HIC (Elizabeth Clery) and LMIC institutions (Neema Kamara, Edouard Nkunzimana) as co-authors, having contributed substantially to data analysis, drafting, and interpretation. They attended the workshops, contributed to evidence synthesis, data collection, analysis and interpretation. The LMIC researchers are based in Ethiopia.

(12) How has gender balance been addressed within the authorship?

The authorship team reflects gender diversity across both HIC and LMIC partners, with balanced representation of men (Edouard Nkunzimana, Merawi Aragaw, Edmund Newman, Radjabu Bigirimana) and women (Femi Nzegwu, Farhana Haque, Elizabeth Clery, Neema Kamara) in leadership and supporting roles.

**Training:**

(13) How has the project contributed to training of LMIC researchers?

The LMIC researchers themselves were technical experts in their respective fields. The project provided structured training mostly in how to conduct systematic reviews.

**Infrastructure:**

(14) How has the project contributed to improvements in local infrastructure?

Physical improvements to local infrastructure were not a component of this study. However, the project has strengthened the research capacity of the local investigators particularly in workshop facilitation and systematic reviews.

**Governance:**

(15) What safeguarding procedures were used to protect local study participants and researchers?

Ethical approvals were obtained from both the London School of Hygiene and Tropical Medicine and Africa CDC. The study objectives, methods, benefits and risks were discussed in detail with relevant ministries/authorities of the African Union Member States participating in the study via online meetings for approval at the inception stage. Ethical clearances were additionally sought from relevant bodies in the case study countries (Nigeria and Namibia). All study activities followed safeguarding procedures to protect participants and research staff, including informed consent, confidentiality protocols and secure data handling.
